# Supplementary material for: AQPX-cluster aquaporins and aquaglyceroporins are asymmetrically distributed in trypanosomes
Source: Commun Biol. 2021 Aug 10;4:953. doi: 10.1038/s42003-021-02472-9 (PMC8355241; doi:10.1038/s42003-021-02472-9)
Supplement: Supplementary file 2 — Supplementary Information [file 42003_2021_2472_MOESM2_ESM.pdf]

Communications Biology Supplementary Information

Article Title: **AQPX-cluster aquaporins and aquaglyceroporins are asymmetrically distributed in trypanosomes**

Authors: Fiorella Carla Tesan, Juan Ramiro Lorenzo Lopez, Karina Alleva, Ana Romina Fox.

Supplementary Results and Discussion

Bacterial AQPs from the AQPX$_{SSN}$ cluster represent a bacterial MIP grade

We explored uncharacterized bacterial MIPs from the subcluster AQPX found within the SSN results. For this, we performed a phylogenetic analysis including bacterial MIPs from the AQPX subcluster of our SSN and MIPs from the prokaryotic grades AqpN, AqpM, Glp, and AqpZ. The phylogenetic tree exposed the already described segregation between prokaryotic Glp, AqpM, AqpN, and AqpZ grades[1,2] (Supplementary Figure 1). And the prokaryotic MIPs of the AQPX cluster of the SSN composed a fifth well-supported grade (AqpX). The phylogeny shows that AqpZ, AqpN, and AqpX are more closely related between them and distant from Glp and AqpM (this is coherent with other published data[1,2]).

The predominant bacteria phyla in each subcluster (AQP, GLP, or AQPX) of our SSN is different (Supplementary Figure 2). Even though, AqpXs do not seem to be characteristic of a specific Bacteria phylum. In some genomes, we found AqpX to be coded together with Glps and AqpZs. To further describe these bacterial AqpX characteristics, we also explored their primary sequence regarding typical MIP residues. The two NPAs were conserved, but the selectivity filter residues had specific identities in each phylogenetic bacterial MIP grade (Supplementary Figure 1). Typical arginine (R) in loop E and the aromatic amino acid in transmembrane 2 were conserved in all the previously known bacterial MIP (Glp, AqpZ, AqpN, AqpNx, AqpZx, and AqpM), whereas AqpX shows none of those residues. Finally, here we described a prokaryotic MIP grade, the AqpX grade. Still, prokaryotic MIPs diversity seems to be larger. Clusters 2, 4, 7, 8, and 10 of our SSN remain unexplored and it would be of great interest to study them to unravel more unreported characteristics of this large superfamily of channels.

Analysis of Discoba genomes and transcriptomes completeness

BUSCO provides a quantitative estimation of the completeness of the assemblies in terms of expected gene content using as reference an OrthoDB set of "universal" orthologs[3]. BUSCO provides different lineage datasets of orthologs and we tested two of them: eukaryota_odb10 and euglenozoa_odb10. The euglenozoa_odb10 provides high scores (77-100%) only for trypanosomatids and *Bodo saltans*. For the rest of the kinetoplastids, it was more or less the same to use one or the other dataset, whereas for diplonemids and euglenids, the eukaryota_odb10 provides higher scores (Supplementary Data 2). The reason for the bias in the analyses is that the euglenozoa_odb10 was built only with trypanosomatids, *Bodo saltans*, and *Perkinsela* genes. Thus, we reasoned that a good compromise was to consider the BUSCO results obtained using the eukaryota_odb and to consider that the trypanosomatids that are completely sequenced got scores ranging from 40 to 56% using this data set. Therefore, the transcriptomes of Neobodonida and Prokinetoplastina that got scores ranging from 40 to 64% (excluding *Perkinsela* that is known to have a small genome[4]) can be considered representative for these species. Diplonemids and Euglenids scores were over 60% of completeness, being reasonable values for transcriptomes. The genus *Naegleria*, the best known and most studied genus within Heterolobosea, got high scores (over 75% complete). Finally, the *Percolomonas cosmopolitus* strains got scores of 35 and 54%. Then, from these assemblies, any suggestion of absence of genes should be taken with caution.

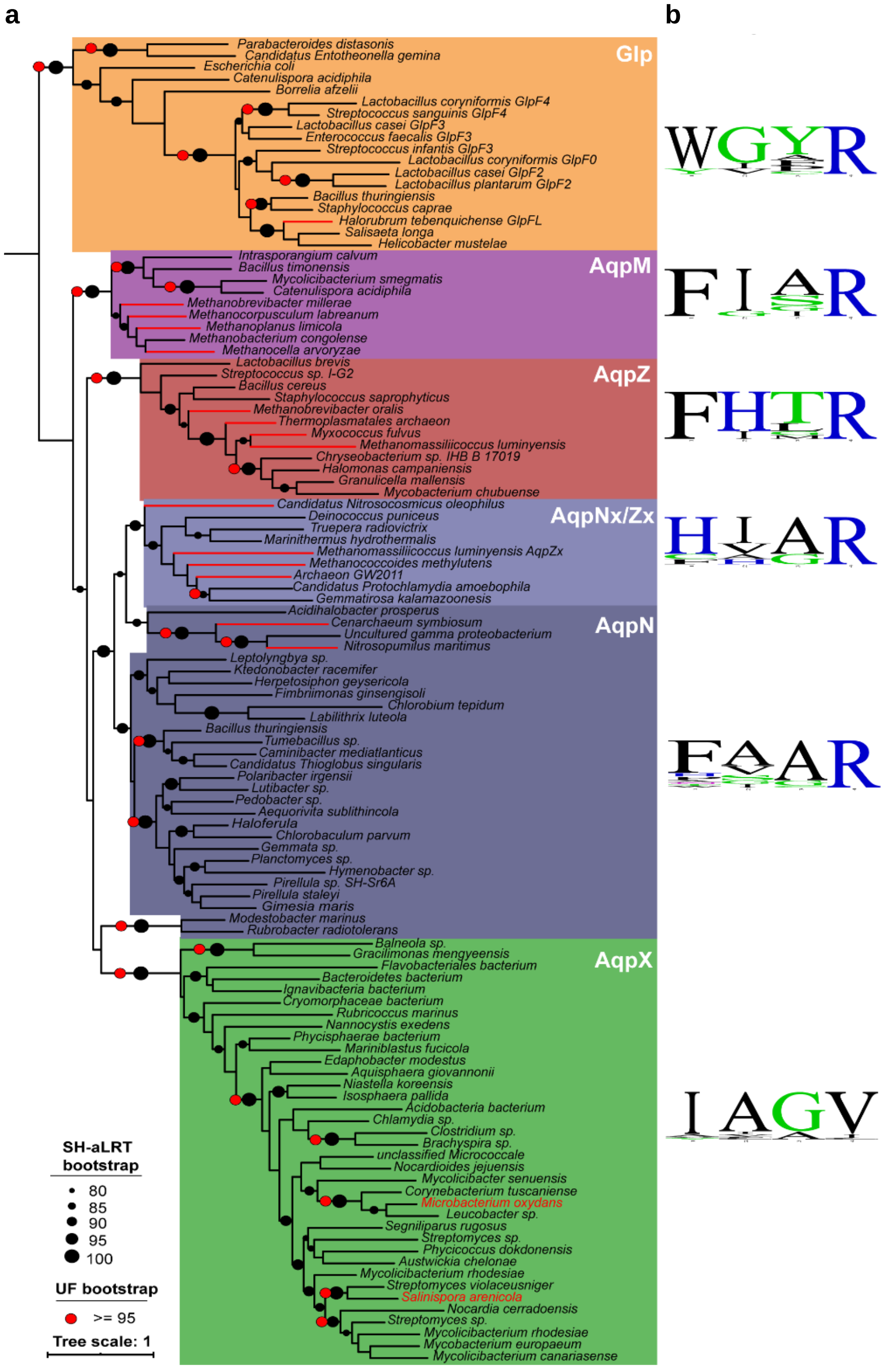

Supplementary Figure 1. Diversity of prokaryotic Major intrinsic proteins (MIPs). a Prokaryotic MIPs phylogenetic tree reconstructed by maximum likelihood. Clades were shaded according to the MIP grade following Pommerrenig et al.[2] classification (i.e., Glp, AqpM, AqpZ, AqpN, and AqpZx/Nx), and the newly proposed grade (AqpX) was shaded in green. Branch support was assessed by the ultrafast bootstrap (UF) approximation with 10,000 replicates and the SH-aLRT with 2,000 replicates. Red branches correspond to Archaea sequences. Names in red correspond to AqpX sequences used in the preliminary phylogenetic tree of Discoba MIPs (Figure 3). **b** Residues of the selectivity filter. A logo graphical view was used to expose the relative abundance of each residue in each position.

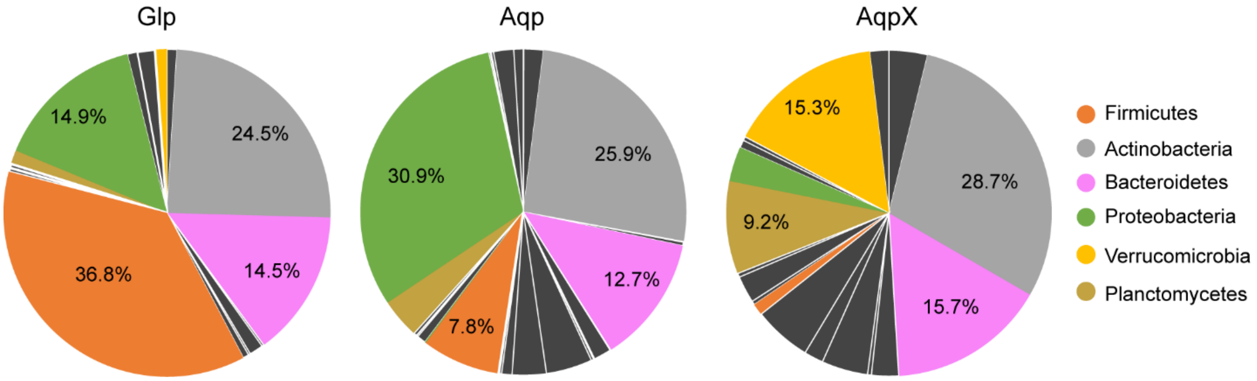

Supplementary Figure 2. Bacteria phyla relative abundance is different in each MIP subcluster of the SSN. Pie charts show the Bacteria phyla composition of each subcluster (Glp, Aqp, and AqpX) of the Cluster 1 of our SSN. In color are those phyla that are one of the four most abundant phyla in at least one subcluster.

Supplementary Table 1. Statistic of the genome assemblies used for the syntenic study of Trypanosomatid MIPs.

Assembly Name	Assembly status	# contigs (≥ 1000 bp)	Total length (≥ 0 bp)	Total length (≥ 1000 bp)	N50*	L50**	GC (%)
TcruziCLBrenerNon-Esmeraldo-like	chromosome	41	32529072	32529072	870934	12	50.71
TbruceiTREU927	chromosome + contigs	131	35826294	35826294	3542885	4	45.49
TgrayiANR4	contigs	2246	20934132	20586005	16942	350	53.97
TtheileriEdinburgh	contigs	252	29602501	29601710	517122	19	40.1
TcongolenseIL3000_2019	chromosome + contigs	375	39170526	39170526	1070342	9	45.98
PconfusumCUL13	supercontigs	975	27575424	26717536	220321	34	61.74
BayalaiB08-376	contigs	546	21553048	21553048	103635	63	54.94
LmajorLV39c5	chromosome + contigs	248	32331530	32074400	1059486	11	59.48
BsaltansLakeKonstanz	supercontigs	2155	39864394	39786871	31876	365	51.79

*sequence length of the shortest contig at 50% of the total genome length.

**smallest number of contigs whose length sum makes up half of genome size.

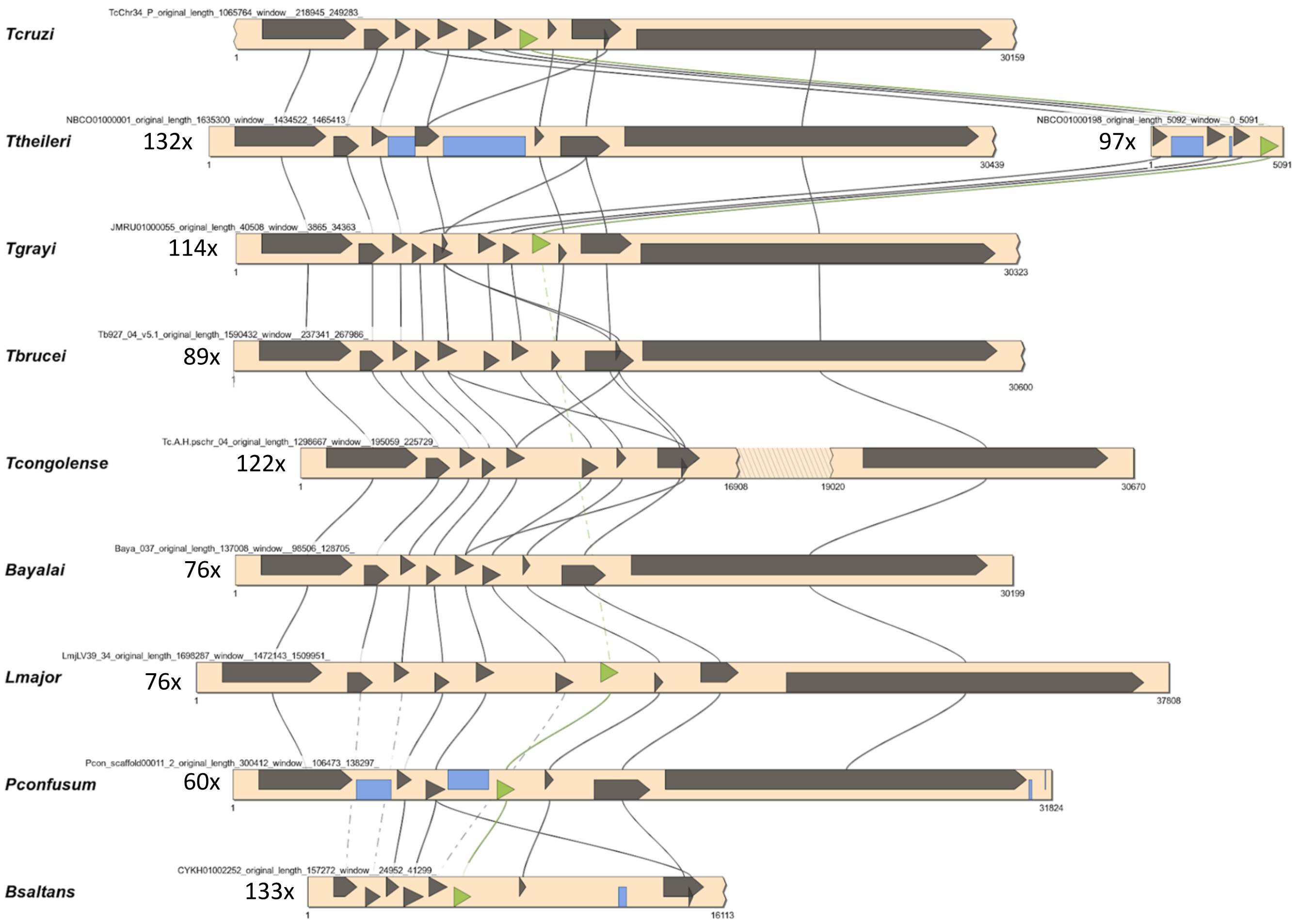

Supplementary Figure 3. Supplementary synteny of *AQPα*. Output of the SimpleSynteny software. *T. cruzi* isoform was used as a reference for syntenic analysis. *AQPα* homolog genes are shown in green. Neighbor genes are shown in dark grey and connecting lines stand for homology. Species names are shown at the right and numbers in contig show end and beginning of the breaks in bp. The average region coverage is reported next to the assemblies. Blue squares denote Ns regions.

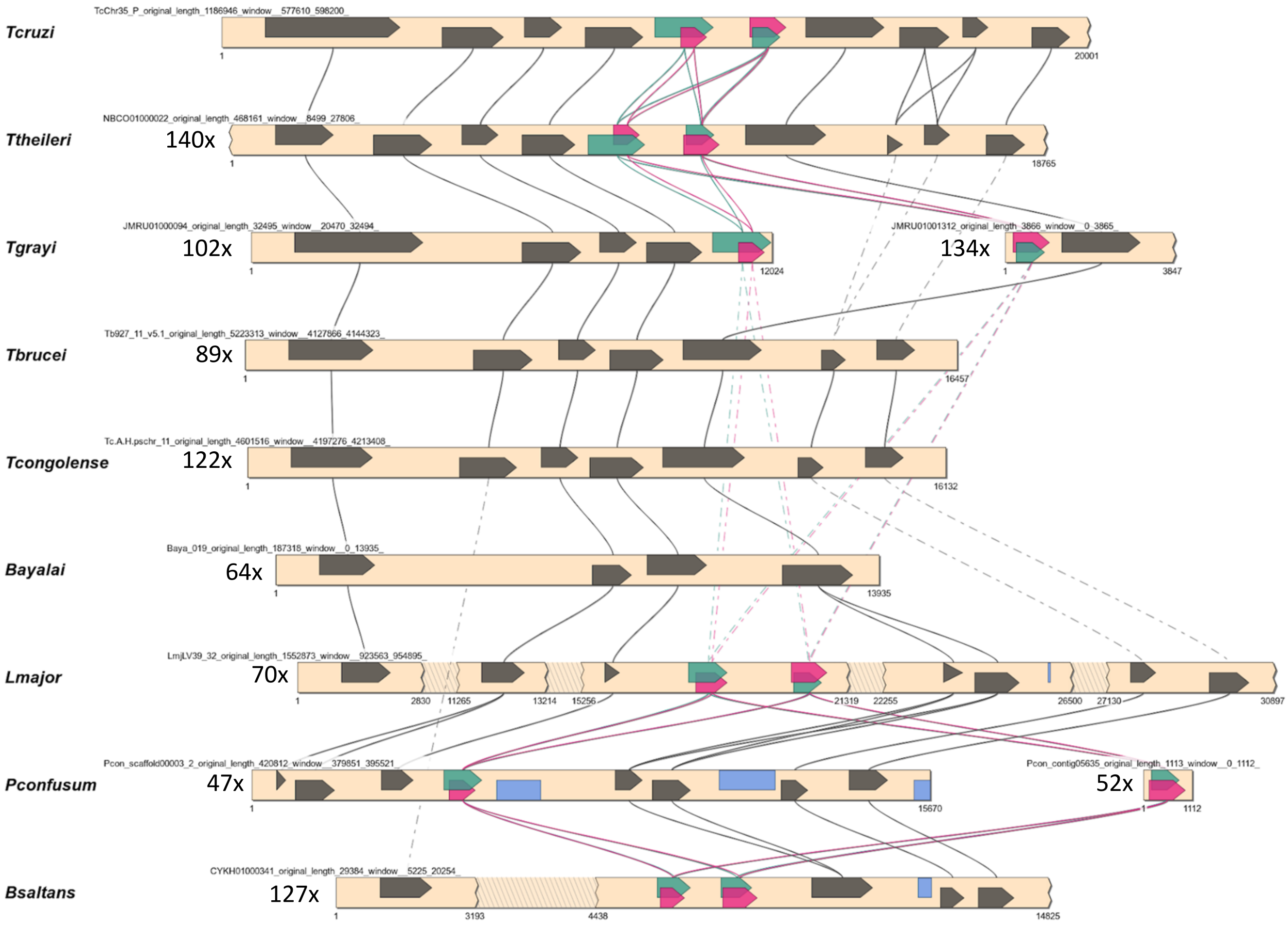

Supplementary Figure 4. Supplementary synteny of *AQPβ* and *AQPδ*. Output of the SimpleSynteny software. *T. cruzi* isoforms were used as a reference for syntenic analysis. *AQPβ* and *AQPδ* homolog genes are shown in cyan and pink, respectively. Neighbor genes are shown in dark grey and connecting lines stand for homology. Species names are shown at the right and numbers in contig show end and beginning of the breaks in bp. Because *AQPβ* and *δ* share identity over the E-value cutoff gene, genes look overimposed in each genome. The average region coverage is reported next to the assemblies. Blue squares denote Ns regions.

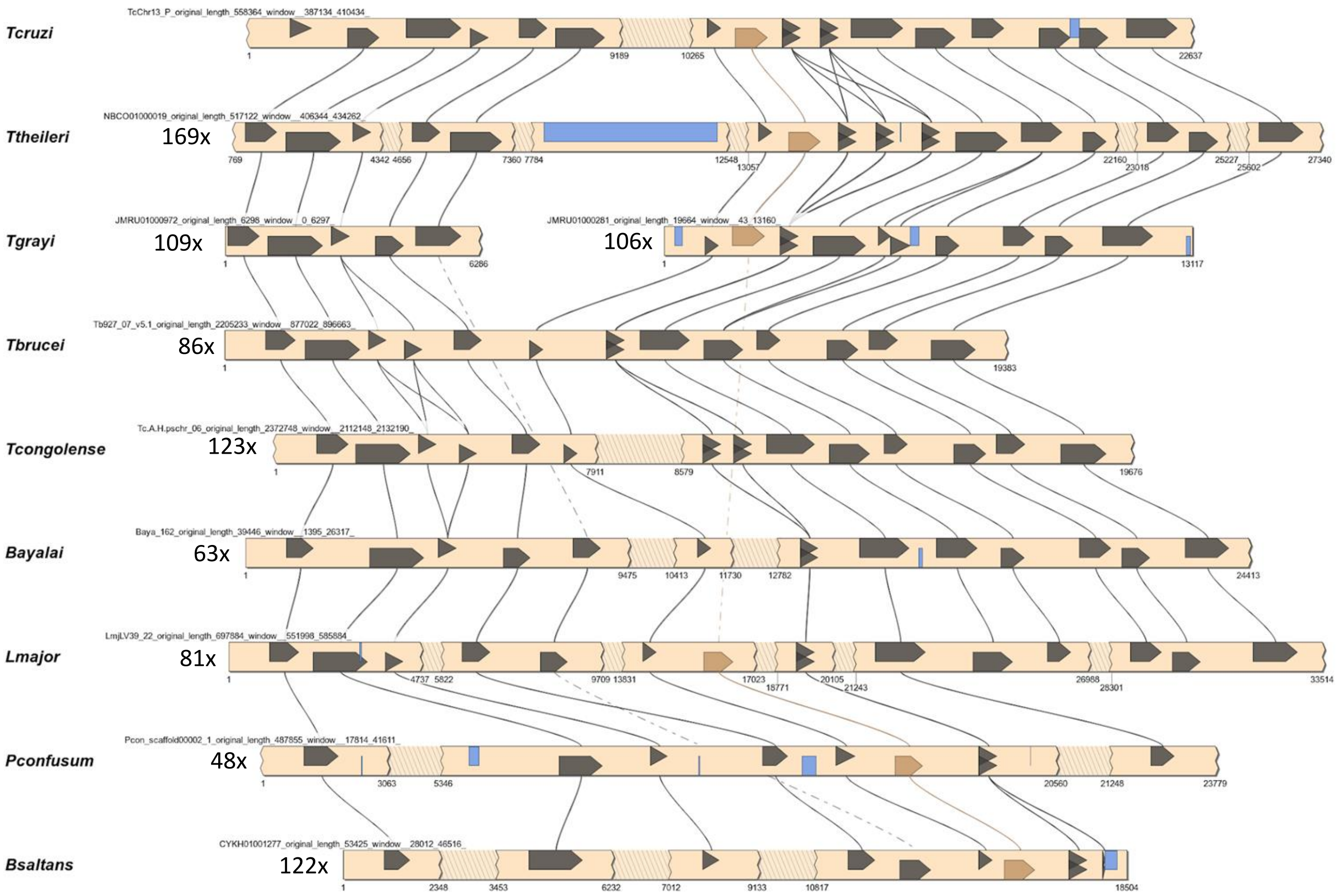

Supplementary Figure 5. Supplementary synteny of *AQPγ*. Output of the SimpleSynteny software. *T. cruzi* isoform was used as a reference for syntenic analysis. *AQPγ* homolog genes are shown in light brown. Neighbor genes are shown in dark grey and connecting lines stand for homology. Species names are shown at the right and numbers in contig show end and beginning of the breaks in bp. The average region coverage is reported next to the assemblies. Blue squares denote Ns regions.

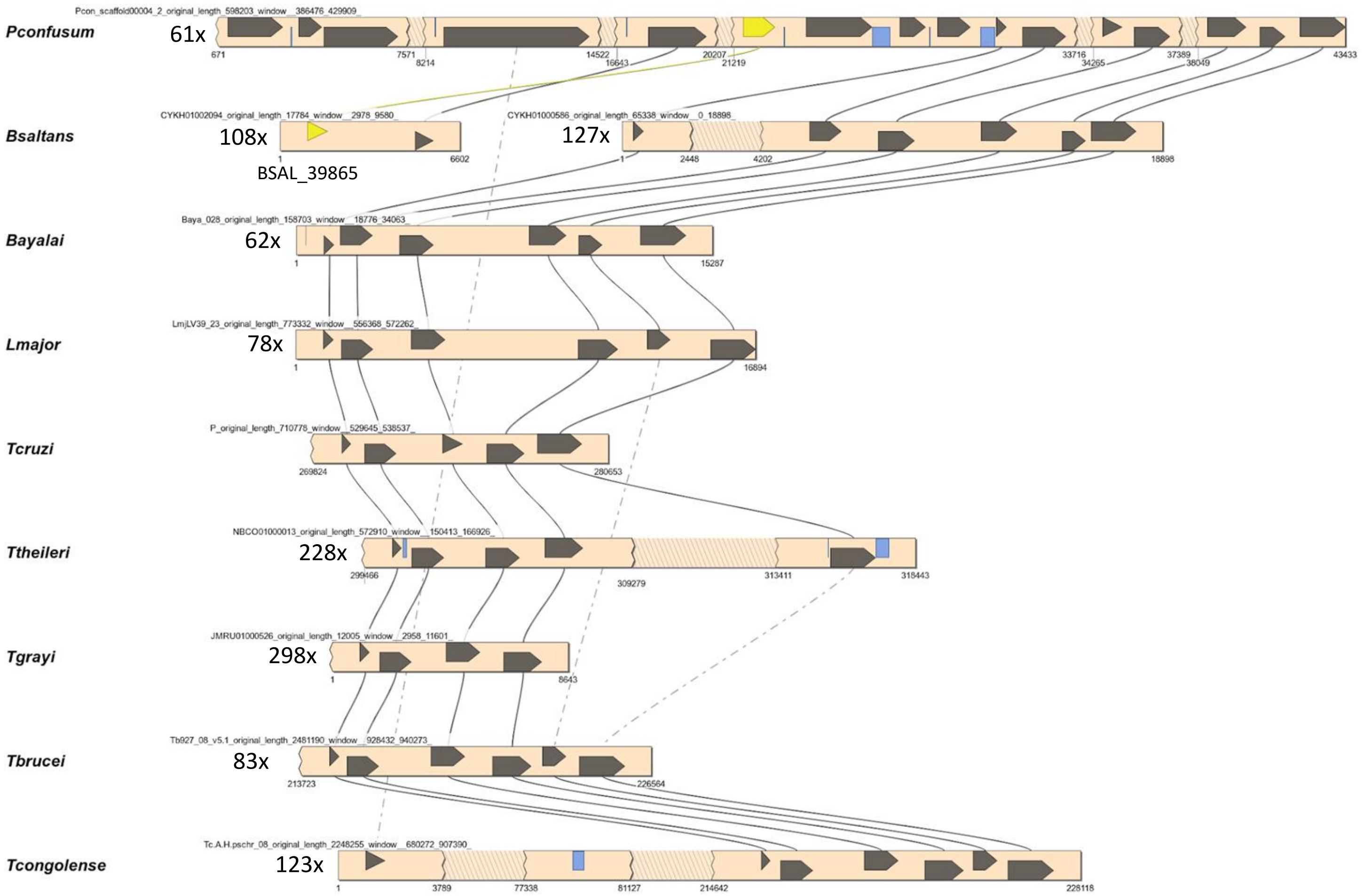

Supplementary Figure 6. Supplementary synteny of coding region of AQPX, PCon_20620 of *Paratrypanosoma confusum.* Output of the SimpleSynteny software. *Pcon_20620* was used as a reference for syntenic analysis and homologs genes are shown in yellow. Neighbor genes are shown in dark grey and connecting lines stand for homology. Species names are shown at the right and numbers in each contig show the end and beginning of the breaks in bp. The average region coverage is reported next to the assemblies. Blue squares denote Ns regions.

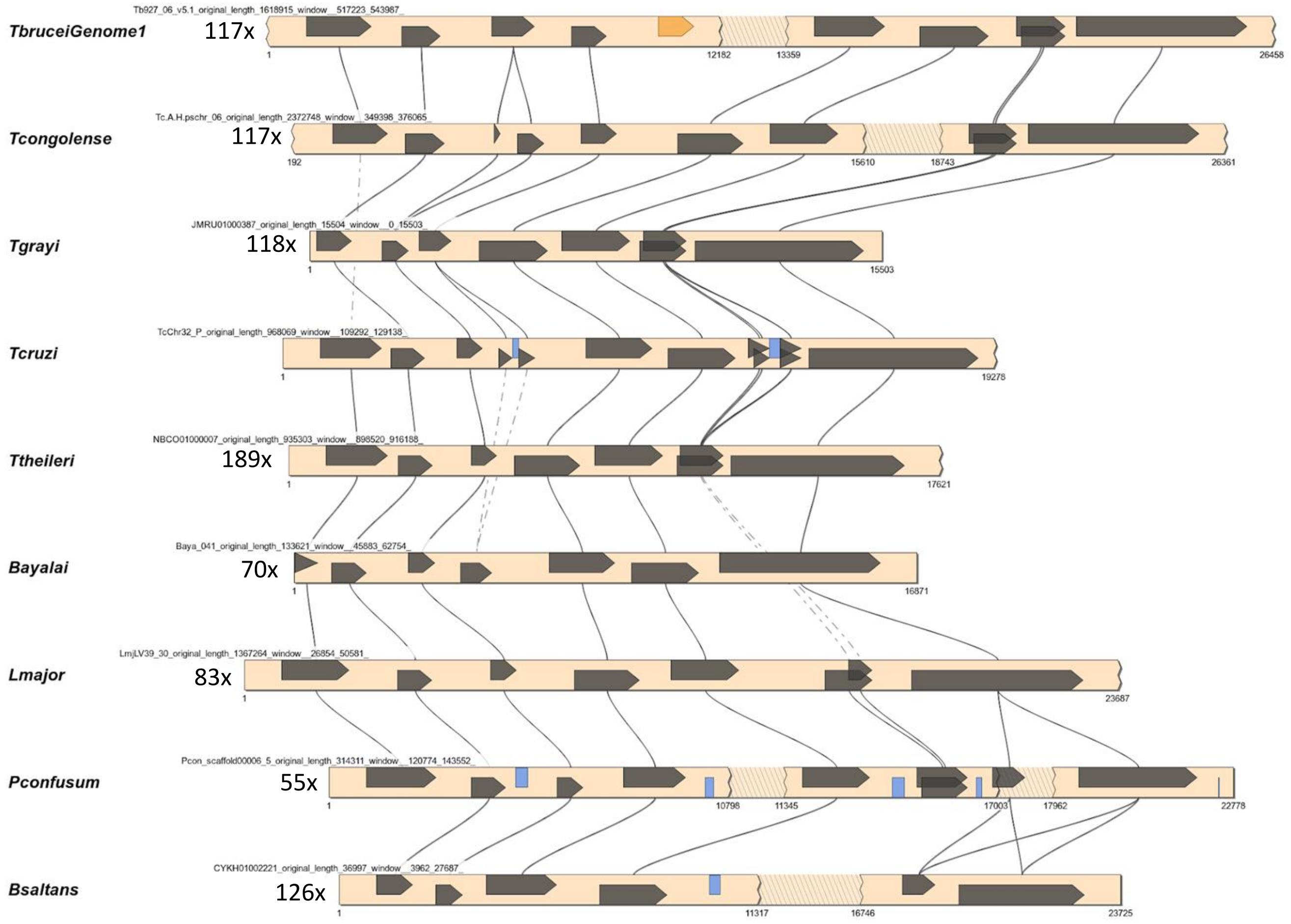

Supplementary Figure 7. Supplementary synteny of *TbAQP1*. Output of the SimpleSynteny software. *T. brucei* isoform was used as a reference for syntenic analysis. *TbAQP1* homolog genes are shown in dark yellow. Neighbor genes are shown in dark grey and connecting lines stand for homology. Species names are shown at the right and numbers in each contig show the end and beginning of the breaks in bp. The average region coverage is reported next to the assemblies. Blue squares denote Ns regions.

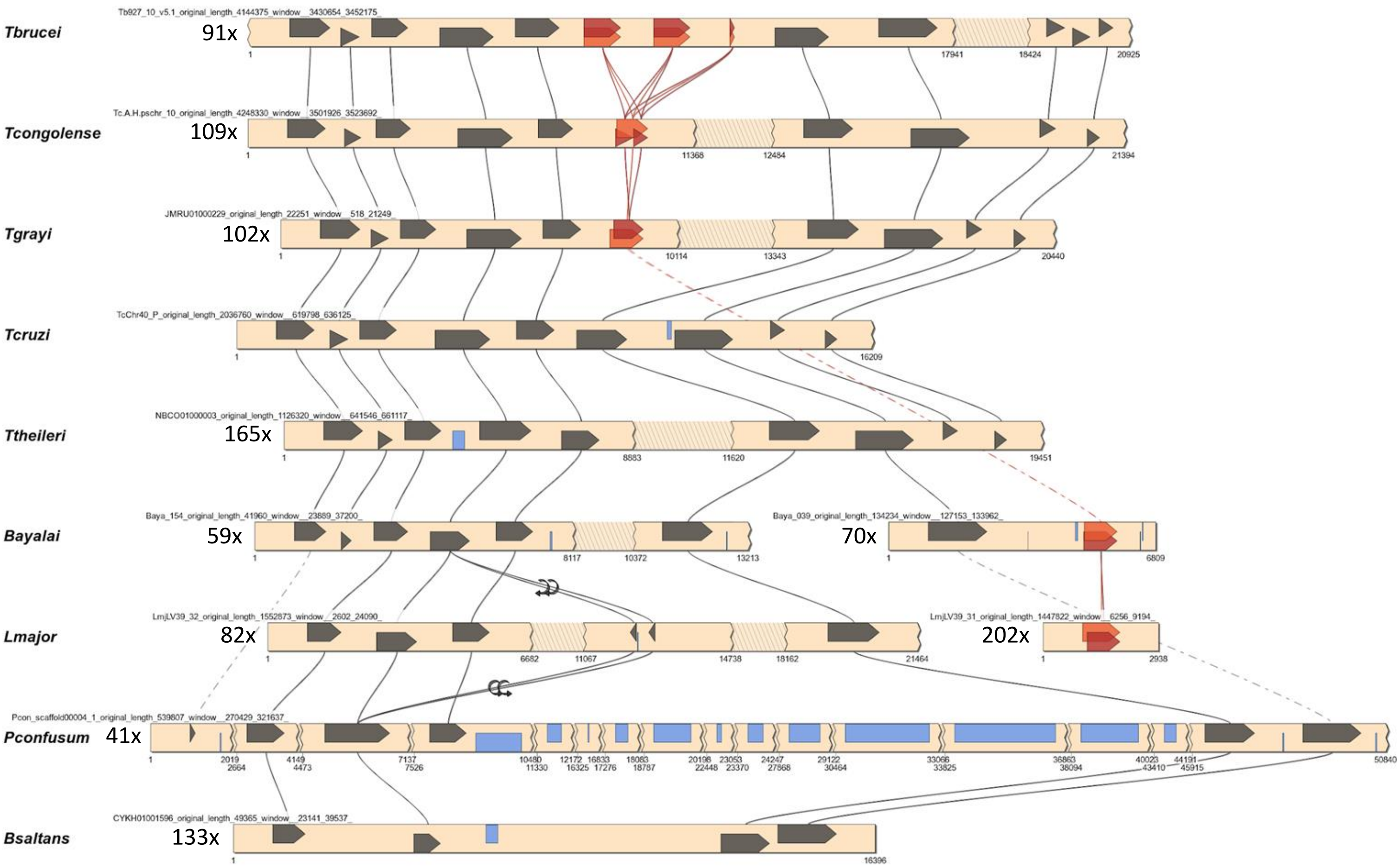

Supplementary Figure 8. Supplementary synteny of *TbAQP2-3*. Output of the SimpleSynteny software. *T. brucei* isoform was used as a reference for syntenic analysis. *TbAQP2* and *TbAQP3* homolog genes are shown in brown and orange, respectively. Neighbor genes are shown in dark grey and connecting lines stand for homology. Species names are shown at the right and numbers in contig show end and beginning of the breaks in bp. Because *TbAQP2* and *TbAQP3* share identity over the E-value cutoff gene, genes look overimposed in each genome. The average region coverage is reported next to the assemblies. Blue squares denote Ns regions.

Supplementary References

1. Finn, R. N., Chauvigné, F., Hlidberg, J. B., Cutler, C. P. & Cerdà, J. The lineage-specific evolution of aquaporin gene clusters facilitated tetrapod terrestrial adaptation. *PLoS One* **9**, 1–38 (2014).

2. Pommerrenig, B. *et al.* Functional evolution of nodulin 26-like intrinsic proteins: from bacterial arsenic detoxification to plant nutrient transport. *New Phytol.* **225**, 1383–1396 (2020).

3. Simão, F. A., Waterhouse, R. M., Ioannidis, P., Kriventseva, E. V. & Zdobnov, E. M. BUSCO: Assessing genome assembly and annotation completeness with single-copy orthologs. *Bioinformatics* **31**, 3210–3212 (2015).

4. Tanifuji, G. *et al.* Genome sequencing reveals metabolic and cellular interdependence in an amoeba- kinetoplastid symbiosis. 1–13 (2017) doi:10.1038/s41598-017-11866-x.
