# Supplementary material for: AQPX-cluster aquaporins and aquaglyceroporins are asymmetrically distributed in trypanosomes
Source: Commun Biol. 2021 Aug 10;4:953. doi: 10.1038/s42003-021-02472-9 (PMC8355241; doi:10.1038/s42003-021-02472-9)
Supplement: Supplementary file 3 — Description of Additional Supplementary Files [file 42003_2021_2472_MOESM3_ESM.pdf]

Description of Additional Supplementary Files

File name: Supplementary Data 1.

Description: MIP prokaryotic sequences. List of the prokaryotic MIP sequences used in this work to construct the prokaryotic phylogenetic tree. The file includes species names, database identifier, the source of the sequence, the MIP grade, and the residues of the selectivity filter of each channel.

File name: Supplementary Data 2.

Description: Genomes and Transcriptomes of Discoba organisms analyzed in this study. The file includes for each organism the target data type (genome, transcriptome, proteome), the database identifier and the BUSCO analyses results using two different databases (eukaryota_odb10 and euglenozoa_odb10).

File name: Supplementary Data 3.

Description: Parabodo caudatus, Procryptobia sorokini and PhF-6 MIPs. Analysis of the transcriptome assemblies in which these organisms were present to retrieve their MIPs. The protein sequences finally assigned to each of these organisms are provided.

File name: Supplementary Data 4.

Description: MIP identifiers and position in the preliminary phylogenetic tree. The file includes species names, the number of MIP in each species, the database identifier of each isoform and their position in the Discoba preliminary tree.

File name: Supplementary Data 5.

Description: Identity matrix of proteins (Discoba MIPs). The multiple sequence alignment used for the Discoba preliminary tree was also used to calculate the amino acid sequence identity using Bioedit.

File name: Supplementary Data 6.

Description: Key MIP residues, solutes permeation and localization.

File name: Supplementary Data 7.

Description: Identity matrix of proteins (AQPX). The multiple sequence alignment used for the Discoba AQPX tree was also used to calculate the amino acid sequence identity using Bioedit.

File name: Supplementary Data 8.

Description: Coverage of the regions used for synteny analysis. Analyzed genome regions

for each MIP, method of coverage calculation and datasets used for mapping.

File name: Supplementary Data 9.

Description: Sequence Similarity Network of the MIP superfamily. Source data for the SSN generated using the EFI-EST server. The network is in .xgmml format.

File name: Supplementary Data 10.

Description: Prokaryotic MIP tree file. Phylogenetic tree built by maximum likelihood using IQ-TREE in NEWICK format.

File name: Supplementary Data 11.

Description: Discoba supergroup MIP tree file. Phylogenetic tree built by maximum likelihood using IQ-TREE in NEWICK format.

File name: Supplementary Data 12.

Description: Discoba AQPX tree file. Phylogenetic tree built by maximum likelihood using IQ-TREE in NEWICK format.
