# Supplementary material for: AQPX-cluster aquaporins and aquaglyceroporins are asymmetrically distributed in trypanosomes
Source: Commun Biol. 2021 Aug 10;4:953. doi: 10.1038/s42003-021-02472-9 (PMC8355241; doi:10.1038/s42003-021-02472-9)
Supplement: Supplementary file 16 — Reporting Summary [file 42003_2021_2472_MOESM16_ESM.pdf]

nature research

Corresponding author(s): Karina Alleva and Ana Romina Fox

Last updated by author(s): Jul 13, 2021

Reporting Summary

Nature Research wishes to improve the reproducibility of the work that we publish. This form provides structure for consistency and transparency in reporting. For further information on Nature Research policies, see our Editorial Policies and the Editorial Policy Checklist.

Statistics

For all statistical analyses, confirm that the following items are present in the figure legend, table legend, main text, or Methods section.

n/a	Confirmed	
x		The exact sample size (*n*) for each experimental group/condition, given as a discrete number and unit of measurement
x		A statement on whether measurements were taken from distinct samples or whether the same sample was measured repeatedly
	x	The statistical test(s) used AND whether they are one- or two-sided<br>*Only common tests should be described solely by name; describe more complex techniques in the Methods section.*
x		A description of all covariates tested
x		A description of any assumptions or corrections, such as tests of normality and adjustment for multiple comparisons
x		A full description of the statistical parameters including central tendency (e.g. means) or other basic estimates (e.g. regression coefficient) AND variation (e.g. standard deviation) or associated estimates of uncertainty (e.g. confidence intervals)
x		For null hypothesis testing, the test statistic (e.g. *F*, *t*, *r*) with confidence intervals, effect sizes, degrees of freedom and *P* value noted<br>*Give P values as exact values whenever suitable.*
x		For Bayesian analysis, information on the choice of priors and Markov chain Monte Carlo settings
x		For hierarchical and complex designs, identification of the appropriate level for tests and full reporting of outcomes
x		Estimates of effect sizes (e.g. Cohen's *d*, Pearson's *r*), indicating how they were calculated

Our web collection on statistics for biologists contains articles on many of the points above.

Software and code

Policy information about availability of computer code

Data collection	The sequence assemblies from Butenko et al. (2020) were provided by Dr. Lukeš lab. Blechomonas ayalai SRAs were provided by Dr. Vyacheslav Yurchenko. The rest of the data was downloaded from public available databases, as stated in the Methods section: UniProt (version 2020-02), TriTrypDB, NCBI and iMicrobe.
Data analysis	All software used in this study has been listed in the Methods section:<br><br>EFI-EST server (Option D) --- to built the sequence similarity networks<br>CD-HIT --- to cluster sequences by identity percentage<br>Cytoscape 3.8 --- to visualize the sequence similarity networks<br>MAFFT v7 --- to align sequences<br>TrimALv1.2rev59 --- to trim MSA<br>rnaSPAdes assembler for RNA-Seq data (Galaxy Version 3.9.0.1) --- to assembly Sequence Read Archives<br>SMART (Simple Modular Architecture Research Tool) --- to corroborate protein domain architectures<br>IQ-TREE v1.6.10 and v2.0-rc2 --- to perform the phylogenetic analyses<br>Interactive Tree of Life tool v5--- to visualize phylogenetic trees<br>BLAST+ (version 2.10.1+, 10.1186/1471-2105-10-421) --- MIP sequences finding in transcriptomes and synteny analysis<br>SimpleSynteny software --- Synteny analysis<br>TriTrypDB genome browser --- Synteny analysis<br>Bioedit 7.2.5 --- to visualize MSA<br>BUSCO tool suite v5.0.0 - genome and transcriptomes completeness<br>Quast v5.0.2 --- to calculate assembly status and metrics of the genomes<br>Bowtie2 --- reads mapping to genome assemblies

Samtools --- coverage analysis

For manuscripts utilizing custom algorithms or software that are central to the research but not yet described in published literature, software must be made available to editors and reviewers. We strongly encourage code deposition in a community repository (e.g. GitHub). See the Nature Research guidelines for submitting code & software for further information.

Data

Policy information about availability of data

All manuscripts must include a data availability statement. This statement should provide the following information, where applicable:

- Accession codes, unique identifiers, or web links for publicly available datasets
- A list of figures that have associated raw data
- A description of any restrictions on data availability

Accession codes, unique identifiers and web links for publicly available datasets are listed in the supplementary data. The datasets generated and/or analyzed during the current study are available from the corresponding author on reasonable request (SRA assemblies, sequences, MSA, sequence identity of the SSN clusters).

Field-specific reporting

Please select the one below that is the best fit for your research. If you are not sure, read the appropriate sections before making your selection.

☐ Life sciences ☐ Behavioural & social sciences ☒ Ecological, evolutionary & environmental sciences

For a reference copy of the document with all sections, see nature.com/documents/nr-reporting-summary-flat.pdf

Ecological, evolutionary & environmental sciences study design

All studies must disclose on these points even when the disclosure is negative.

Study description	We built a sequence similarity network to analyze kinetoplastid sequences' relationship with the other members of the MIP superfamily. We found a distanced and uncharacterized subcluster, which we further studied by phylogenetic inferential methods. We used a maximum-likelihood inference approach with comprehensive inclusion of all MIPs from Discoba organisms. The preliminary phylogeny of this protein superfamily revealed an expanded MIP family among Kinetoplastids that we named AQPX. We also unveiled the asymmetric MIP repertoire among kinetoplastids. Comprehensive inclusion of Kinetoplastid sequences in the AQPX phylogeny built by the maximum likelihood method suggested this family expanded in the metakinetoplastina common ancestor before the origin of parasitism. Genome synteny comparisons among trypanosomatids showed specific losses of AQPX genes (by accumulating mutations or loss of the specific genomic region) in African trypanosomes. Whereas American trypanosomes lost the GLP gene, which we proposed was acquired in the trypanosomatids common ancestor after Paratrypanosoma confusum branched. AQPXs carry novel amino acids in the crucial MIP positions (related to their putative permeation function) as exposed by the primary sequence analysis.
Research sample	SSN accessions were retrieved from the Uniprot database, collecting sequences from the whole MIP superfamily (tagged as PF00230) to place kinetoplastid MIPs in the sequence-function space of the superfamily. To reconstruct evolutionary relationships of MIPs from the Discoba supergroup, we included Jakobid, Heterolobosean, and Euglenozoan sequences.The preliminary phylogenetic tree was built with 132 MIP sequences from Discoba organisms listed in supplementary data. AQPX phylogenetic tree was built with 100 MIP sequences (listed in supplementary data). Synteny analysis included organisms from Trypanosomatida order and Bodo saltans to unravel MIP asymmetry in the mentioned order.
Sampling strategy	We searched for MIP sequences within the complete Discoba supergroup. To avoid over representation of trypanosomatid MIPs in the preliminary tree, only representative trypanosomatids were included. The same procedure was followed to select MIPs for the tree of AQPXs, not all Leishmania species were included in the analyses.
Data collection	Data colection (MIP sequences) was performed from UniProt (version 2020-02), TriTrypDB, NCBI, iMicrobe, Trascriptome assemblies provided by Dr. J Lukeš and our own assemblies. Data collection was performed by BLAST and MIP domain architectures was analysed using the tool SMART. Dr. Vyacheslav Yurchenko provided Blechomonas ayalai SRAs.
Timing and spatial scale	Not applicable.
Data exclusions	For SSN, sequences with less than 200 or more than 500 amino acids were excluded. For phylogenetic analysis, partial sequences from well-represented taxa were excluded as stated in Supplementary data.
Reproducibility	Sequence database and filtration procedures for the SSN construction are fully described in the manuscript. Sequence accession and sources are listed in the supplementary data. Software versions and parameters used were described in the methods.
Randomization	For phylogenetic analyses, the evolutionary relationships among sequences were inferred by using the maximum likelihood (ML) method. Branch support was calculated with two test: the ultrafast bootstrap test (10,000 iterations) and the Shimodaira-Hasegawa approximate likelihood ratio test (1,000 or 2,000 iterations).

Blinding	Not applicable.

Did the study involve field work? ☐ Yes ☒ No

Reporting for specific materials, systems and methods

We require information from authors about some types of materials, experimental systems and methods used in many studies. Here, indicate whether each material, system or method listed is relevant to your study. If you are not sure if a list item applies to your research, read the appropriate section before selecting a response.

Materials & experimental systems

n/a	Involved in the study
☒	☐ Antibodies
☒	☐ Eukaryotic cell lines
☒	☐ Palaeontology and archaeology
☒	☐ Animals and other organisms
☒	☐ Human research participants
☒	☐ Clinical data
☒	☐ Dual use research of concern

Methods

n/a	Involved in the study
☒	☐ ChIP-seq
☒	☐ Flow cytometry
☒	☐ MRI-based neuroimaging
